# Effect of Chromium(VI) Toxicity on Enzymes of Nitrogen Metabolism in Clusterbean (*Cyamopsis tetragonoloba* L.)

**DOI:** 10.1155/2014/784036

**Published:** 2014-03-18

**Authors:** Punesh Sangwan, Vinod Kumar, U. N. Joshi

**Affiliations:** ^1^Department of Biochemistry, CCS Haryana Agricultural University, Hisar 125001, India; ^2^Department of Biochemistry, G. B. Pant University of Agriculture and Technology, Pantnagar 263145, India

## Abstract

Heavy metals are the intrinsic component of the environment with both essential and nonessential types. Their excessive levels pose a threat to plant growth and yield. Also, some heavy metals are toxic to plants even at very low concentrations. The present investigation (a pot experiment) was conducted to determine the affects of varying chromium(VI) levels (0.0, 0.5, 1.0, 2.0, and 4.0 mg chromium(VI) kg^−1^ soil in the form of potassium dichromate) on the key enzymes of nitrogen metabolism in clusterbean. Chromium treatment adversely affect nitrogenase, nitrate reductase, nitrite reductase, glutamine synthetase, and glutamate dehydrogenase in various plant organs at different growth stages as specific enzyme activity of these enzymes decreased with an increase in chromium(VI) levels from 0 to 2.0 mg chromium(VI) kg^−1^ soil and 4.0 mg chromium(VI) kg^−1^ soil was found to be lethal to clusterbean plants. In general, the enzyme activity increased with advancement of growth to reach maximum at flowering stage and thereafter decreased at grain filling stage.

## 1. Introduction

Excessive levels of heavy metals in agricultural lands constitute an increasingly serious threat not only for intact plant growth and yield but also for the environment and human health [1]. The toxicity of plants due to heavy metals, particularly on agricultural economic crops, presents a challenge to plant scientists concerned with yield and quality in crop production [2]. These metals retard farming efficiency and destroy the health of the plants and animals [3]. Some heavy metals such as chromium (Cr), lead (Pb), mercury (Hg), and cadmium (Cd), especially in large amounts, could affect growth and productivity of plants [4]. They are usually accumulated due to unplanned municipal waste disposal, mining, use of extensive pesticides, and chemical fertilizers [5].

Nowadays, contamination of the environment by Cr has become a major concern and its toxicity to plants depends on its valence state with Cr(VI) being highly toxic and mobile than Cr(III) [6]. Cr(III) occurs naturally in the environment and is an essential nutrient, whereas Cr(VI) is generally produced by industrial processes [7]. It is found in all parts of the environment including air, water, rocks, and soil [5]. The anthropogenic sources of Cr input are electroplating, mining processing, wood preservation, iron and steel production, pigment manufacture, power station industry, leather tanning, textile preservation, textile printing, paint and porcelain manufacturing, and during combustion of coal and petroleum [8, 9]. Significant quantities of Cr are also added to soil by the disposal of fly ash as well as phosphatic fertilizer application [10].

There are a number of factors which influence plant growth, among them nitrogen is one of the most essential elements and its availability in soil is one of the key factors for determining plant growth and productivity [10]. Nitrogen is considered to be a vital macronutrient for plants and has a role in metabolism [11]. It is incorporated in plant tissues as nucleotides, nucleic acids, coenzymes, vitamins, pigments, alkaloids, amines, and other compounds [12]. It is structural component of wide array of vital biomolecules, such as amino acids, proteins, nucleotides, porphyrins, several coenzymes, chlorophylls, vitamins, and glycosides. On average, proteins and nucleic acids contain about 15% and 13% nitrogen, respectively [12]. Hence, the maintenance of proper N-metabolism of plant can lead to proper growth and development of plant. The nitrogen metabolism and enzymology are related to the selection of high yielding crops. It has been shown that enzymes of nitrogen metabolism are severely affected by different metals and thus reduced crop yields. The nitrogen metabolism is of central importance under stressful conditions [13].

According to Dixit et al. [14], Cr is toxic to plants and interferes with several metabolic processes as exhibited by reduced seed germination or early seedling development, induced chlorosis in young leaves, reduced pigment content, damaged root cells, impaired photosynthesis, altered enzymatic function, stunted growth, and plant death [15]. In India, Cr(VI) contamination is a major problem around various industries using Cr compounds, which causes considerable negative impact on crop production [16].

Clusterbean (*Cyamopsis tetragonoloba *L.) has been grown in India since ancient times for vegetables and fodder purposes. It is an important* kharif* legume commonly known as guar and is cultivated throughout India for its edible pods. Its green pods are used as vegetable, seeds as source of industrial gum, and green plants as fodder and for soil manuring purposes. It has recently assumed great industrial importance due to the presence of gum, that is, galactomannan, in its endosperm which constitutes 25–35% of whole seeds and is highly mucilaginous. Gum is used extensively in paper, mining, explosive, food, pharmaceuticals, cosmetics, textiles, and oil industries [17]. In Haryana (a major guar producing state), Sonepat, Panipat, Dharuhera, Gurgaon, Yamunanagar, Faridabad, and Shahabad are the main industrial areas, where poor plant growth of field crops has been observed. Contamination of soils by heavy metals as a result of human, agricultural, and industrial activities is a major cause of poor plant growth [18]. However, not much work has been reported on the effect of Cr(VI) on guar. Considering the above facts and the importance of nitrogen in plant growth, the present study was carried out to explore the effect of Cr(VI) on nitrogen metabolism in clusterbean which will be beneficial to the understanding of Cr(VI) induced changes in plant nitrogen metabolism.

## 2. Material and Methods

### 2.1. Chemicals, Reagents, and Soil

The chemicals and reagents used during the present investigation were of analytical grade. A nutrient deficient loamy sand soil from the Regional Research Station, Gangwa block of Hisar district, was used in the present study. The characteristics of soil were pH (1 : 2) 8.50; organic carbon, 0.22%; N, 4.0 mg kg^−1^ soil; P, 13.0 mg kg^−1^ soil; K, 163 mg kg^−1^ soil; Zn^2+^, 0.61 mg kg^−1^ soil; Fe^2+^, 0.9 mg kg^−1^ soil; Cu^2+^, 0.18 mg kg^−1^ soil; Mn^2+^, 3.6 mg kg^−1^ soil; EC, 1.5; CaCO_3_ 3.5%; and Cr^2+^, 0.01 mg kg^−1^ soil; texture—sandy loam.

### 2.2. Plant Growth and Environmental Conditions

Seeds of clusterbean (*Cyamopsis tetragonoloba *(L.) Taub.) cv HG 2–20 were procured from Forage Section, Department of Genetics and Plant Breeding, C.C.S. Haryana Agricultural University, Hisar, and raised in pots filled with 5 kg of sandy loam soil in a naturally lit net house. The temperature and relative humidity during the experiment ranged from 11.0 to 35.6°C and 34.5 to 95.2%, respectively. The light intensity ranged from 36100 to 84000 lux. The pots were lined with polythene bags and the soil in each pot was treated with different levels of Cr in the form of potassium dichromate at concentrations of 0.0, 0.5, 1.0, 2.0, and 4.0 mg kg^−1^ soil. The seeds were surface sterilized with mercuric chloride and after proper washing with distilled water, they were inoculated with* Rhizobium *culture. An equal amount of nutrient solution was supplied at weekly intervals to each pot. The plants were irrigated with equal quantities of tap water as and when required. Plant samples from each treatment were collected at vegetative (30 DAS), flowering (50 DAS), and grain filling stages (65 DAS).

### 2.3. Enzyme Activity Measurement

Specific activity in leaves, shoot, and root at different growth stages was measured by standard methods. All observations were measured up to 2.0 mg kg^−1^ soil because plants treated with more than 2.0 mg Cr(VI) kg^−1^ soil concentration did not survive 20 days after sowing.

#### 2.3.1. Sample Extraction for Nitrate Reductase and Nitrite Reductase

One gram of plant tissue was hand homogenized in 10 mL cold phosphate buffer (0.15 M, pH 7.5) containing 1 mM cysteine and 1% (w/v) casein in a previously chilled mortar using acid-washed sand as an abrasive. The homogenate was centrifuged at 10,000 ×g for 30 min at 4°C. The resultant supernatant was taken as the enzyme extract and stored in a refrigerator for enzyme assays and soluble protein estimation.

#### 2.3.2. Sample Extraction for Glutamine Synthetase, Glutamate Dehydrogenase, and Glutamate Synthase

One g of plant tissue was hand homogenized in 10 mL of cold phosphate buffer (0.1 M, pH 7.6) containing 2% polyvinylpyrrolidone, 1% *β*-mercaptoethanol, and 10 mM dithiothreitol in previously chilled mortar using acid-washed sand as an abrasive material [19]. The homogenate was centrifuged at 10,000 ×g for 30 min at 4°C. The resultant supernatant was taken as the enzyme extract and stored in a refrigerator for enzyme assays and soluble protein estimation.

#### 2.3.3. Nitrogenase (E.C.1.7.99.2)

Nitrogenase activity in nodules was measured by acetylene reduction assay as described by Hardy et al. [20]. The roots were separated from the shoots and blotted to remove excess moisture and intact nodules were placed in test tubes (75 mL). A subaseal was placed on the mouth of tubes. An atmosphere of 10% acetylene was created and the tubes were incubated for 2 h at room temperature (28°C). The ethylene so produced was measured by Gas Chromatograph (Hewlett Packard Model 5730) using a dual flame ionizing detector. Nitrogen at a flow rate of 38 mL min^−1^ was used as carrier gas and hydrogen at the flow rate of 25 mL min^−1^ was used as fuel gas. Temperature of oven, detector, and injection port was set at 65, 150, and 100°C, respectively. Standard ethylene (110 ppm) was used for quantification of data. Specific nitrogenase activity expressed as *μ*M C_2_H_4_ produced g^−1^ fresh weight of nodules h^−1^.

#### 2.3.4. Nitrate Reductase (E.C.1.6.6.1)

Nitrate reductase (NR) was assayed by the method suggested by Hageman and Flesher [21]. The assay mixture in a final volume of 2 mL contained (*μ*M) phosphate buffer (pH 7.5), 200; KNO_3_, 20; and NADH, 0.4. The enzymatic reaction was initiated by the addition of 0.5 mL of enzyme extract. A blank without NADH was also run simultaneously. After incubation at 30°C for 15 min, the reaction was terminated by rapidly adding 0.1 mL of 1 M zinc acetate and 1.9 mL of 70% ethanol. The contents were mixed thoroughly and centrifuged at 3000 ×g for 15 min. Two milliliters of supernatant was then removed and transferred to another test tube. One milliliter of 1% sulphanilamide reagent (prepared in 1 N HCl) followed by 1 mL of 0.02% N-1-naphthyl ethylene diamine dihydrochloride was added. After 30 min, the absorbance of the violet color was measured at 540 nm on a spectrophotometer (Systronics 118). The enzyme activity has been expressed as *μ*M of nitrite produced h^−1^ mg^−1^ protein.

#### 2.3.5. Nitrite Reductase (E.C.1.7.7.1)

Nitrite reductase (NiR) activity was measured following the method of Sawhney and Naik [22]. The assay mixture in a final volume of 2 mL contained (*μ*M) phosphate buffer (pH 7.5), 100; NaNO_2_, 1.0; and methylviologen, 0.4 and 0.5 mL of enzyme extract. The reaction was started with the addition of 0.1 mL of sodium dithionite solution (prepared by dissolving 10 mg sodium dithionite in 10 mL of 0.29 M sodium bicarbonate). In a separate tube, sodium bicarbonate without sodium dithionite was run simultaneously as a blank. After incubation for 30 min at 30°C, the reaction was stopped by shaking vigorously. A 0.1 mL volume of the supernatant was aliquoted into a test-tube and added to this was 1.9 mL of distilled water and 1 mL of 1% sulphanilamide reagent (prepared in 1 N HCl) followed by 1 mL of 0.02% N-1-naphthyl ethylene diamine dihydrochloride. After 30 min, the absorbance of violet color was measured at 540 nm on a spectrophotometer. The enzyme activity has been expressed as *μ*M of nitrite reduced h^−1^ mg^−1^ protein.

#### 2.3.6. Glutamine Synthetase (E.C.6.3.1.2)

The activity of glutamine synthetase (GS) was assayed by the method of O'Neal and Joy [23]. The reaction mixture (4 mL) contained 0.1 M Tris-maleate buffer (pH 7.5); 1 M hydroxylamine; 100 mM glutamate; 10 mM ATP; 1 M MgSO_4_; and 0.2 mL of diluted enzyme extract. The reaction was started by adding hydroxylamine and the mixture was incubated for 20 min at 30°C. The reaction was stopped by the addition of 1 mL of FeCl_3_ reagent, prepared by mixing equal volumes of 10% FeCl_3_·6H_2_O in 0.2 M HCl, 24% TCA, and 5% HCl. After 10 min, the protein precipitate was removed by centrifugation. Absorbance of the supernatant was taken at 540 nm and hydroxamic acid concentration was computed using *γ*-glutamyl monohydroxamate (*γ*-GMH) as standard. The results were expressed as *μ*M of *γ*-GMH formed h^*‒*1 ^mg^*‒*1^ protein.

#### 2.3.7. Glutamate Synthase (E.C.1.4.1.14)

Glutamate synthase (GOGAT) activity was measured using the method of Singh and Srivastava [24]. Briefly, the assay mixture contained 0.4 mL 20 mM L-glutamine, 0.4 mL 5 mM 2-oxoglutarate, 1 mM EDTA (added in assay buffer), 0.1 mL 100 mM KCI, 0.6 mL 1 mM NADH, and 0.5 mL of the enzyme preparation in a final volume of 3.0 mL prepared with 25 mM sodium phosphate (pH 7.5). The reaction was started by adding L-glutamine immediately following the enzyme preparation. The decrease in absorbance was recorded for 5 min at 340 nm on a spectrophotometer. The amount of NADH oxidized was calculated from a standard curve of NADH. The results were expressed as *μ*M NADH oxidized h^−1^ mg^−1^ protein.

#### 2.3.8. Glutamate Dehydrogenase (E.C.1.4.1.4)

Glutamate dehydrogenase (GDH) activity was measured following the method of Boland et al. [19]. In brief, the assay mixture (2 mL) contained 14 mM 2-oxoglutarate; 80 mM imidazole-HCl (pH 7.9); 200 mM ammonium acetate; 60 mM NADH; 2 mM ADP; and 0.1 mL enzyme extract. Rate of the reaction was followed by recording the change in absorbance at 340 nm on a spectrophotometer. Background rates were also measured in the absence of ammonium acetate. The enzyme activity was expressed as *μ*M NAD^+^ formed h^−1^ mg^−1^ protein.

### 2.4. Protein Estimation

The soluble protein in the enzyme extract was determined by the method of Lowry et al. [25] followed by itsprecipitation by 20% TCA, centrifugation, and dissolving residue in 0.1 N sodium hydroxide (NaOH) solution.

### 2.5. Statistical Analysis

A two-factorial ANOVA in complete randomized block design was used to confirm the validity of the data using the OPSTAT software available on CCSHAU website homepage (http://hau.ernet.in/opstat.html). The values used in graphs are mean of three replicates and are shown as ± standard error.

## 3. Results and Discussion

### 3.1. Nitrogenase

**Figure 1:**
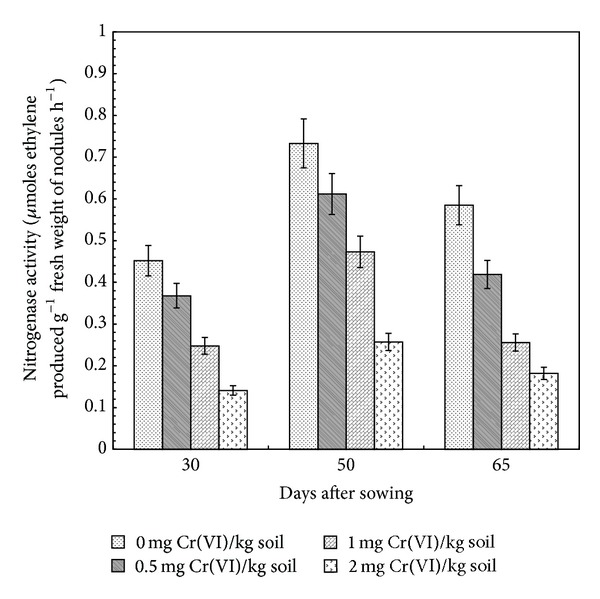
Effect of Cr(VI) on specific nitrogenase activity in nodules of clusterbean plants at different stages of growth.

Nitrogen is one of the major constituents of biological systems. In nature, highly specialized nitrogenase enzymes are expressed by soil bacteria and perform an important function of nitrogen fixation by converting atmospheric nitrogen into ammonia under mild temperature and pressure conditions [26]. In the present study, the gradual and significant decrease in specific nodule nitrogenase activity (SNA) (*μ*M ethylene produced g^−1^ fresh weight of nodules h^−1^  × 10^2^) was observed at different stages of growth in nodules with increasing doses of Cr(VI) concentration from 0.0 to 2.0 mg Cr(VI) kg^−1^ soil (Figure 1). A similar decrease in nitrogen fixation in response to Cr was observed in* Pisum sativum* [27]. At 30 DAS, SNA decreased by 1.3-, 1.9-, and 3.2-fold with respect to control at 0.5, 1.0, and 2.0 mg Cr(VI) kg^−1^ soil, respectively. Maximum decline in SNA was 69% at 65 DAS with 2.0 mg Cr(VI) kg^−1^ soil. Specific nodule nitrogenase activity decreased by 16.5, 35.4, and 65.4% with respect to control at 0.5, 1.0, and 2.0 mg Cr(VI) kg^−1^ soil at 50 DAS, respectively. In control as well as Cr treated plants, SNA increased abruptly from day 30 onwards to evince maximum value at day 50 and after that declined at 65 DAS. According to Balestrasse et al. [28], high cadmium concentration or salt stress resulted in oxidative stress with increased thiobarbituric acid reactive substances content and decreased leghemoglobin level and, consequently, decreased SNA. Decreased SNA may also be due to the effect of heavy metal ions on O_2_ uptake by bacteroid which could result in inhibition of acetylene reduction activity. Bacteroid respiration provides the energy and reducing power that nitrogenase needs for efficient nitrogen fixation [28]. Under a high concentration of metals, the decreased SNA might also be due to decreased nodule number and their biomass [29, 30], induced nodule senescence [28], altered nodule ultrastructure [31], and/or altered plant growth-promoting activities of microorganisms [34].

### 3.2. Nitrate Reductase

**Figure 2:**
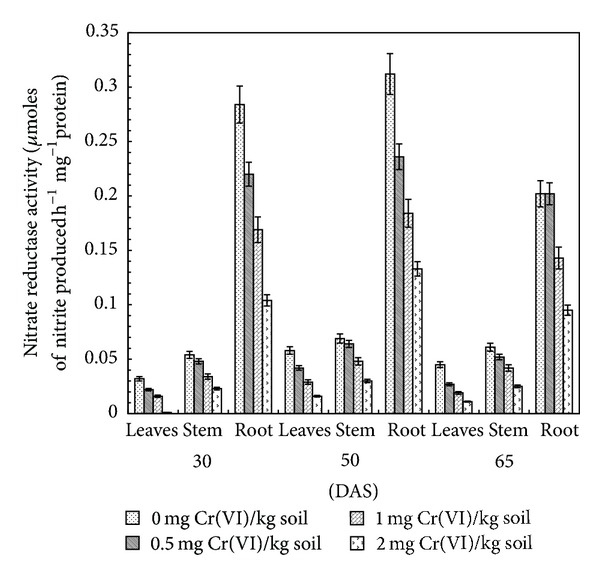
Effect of Cr(VI) on nitrate reductase activity in clusterbean plant parts (leaves, stem, and root) at different stages of growth.

NR is the first enzyme in the process of nitrate reduction and plays an important role in nitrogen assimilation by catalyzing the reduction of nitrate to nitrite. Nitrate is the common source of nitrogen available to plants. Once nitrate is being absorbed by plants it may be reduced in roots, stored in the vacuoles, or transferred to the shoots before being processed. The conversion of nitrate into ammonia is brought about by successive and regulated action of NR and NiR [32]. It is affected by plant development stages and plant parts such as roots and tops and environmental conditions [18]. Cr application in soil was found to have negative effect on the NR specific activity (*μ*M of nitrite produced h^−1^ mg^−1^ protein). The specific activity was decreased by 70.1, 71.9, and 74.9% in leaves, 57.6, 55.9, and 59.2% in stem, and 63.4, 57.3, and 65.0% in root of 2.0 mg Cr(VI) kg^−1^ soil treated plants over control at 30, 50, and 65 DAS, respectively (Figure 2). In each treatment, NR specific activity in various plant parts increased with plant age, attaining the maximum value at 50 DAS, and decreased thereafter at 65 DAS. Root had higher specific activity followed by stem and leaves.

Cr-induced toxicity resulting in reduced activity of NR was also reported in sorghum and Indian mustard [18, 33]. Study on wheat and Indian mustard revealed that lower doses of Cr had stimulatory effects, while higher doses had inhibitory effects on NR activity [33, 34]. A significant increase in NR activity was observed corresponding to Cr concentration in* Brassica juncea *[35]. However, in the present study, no stimulatory effect of low Cr(VI) concentration was observed. The results are in conformity with the observation of Kumar and Joshi [18], where NR specific activity decreased with increase in Cr(VI) concentration. It is suggested that a negative influence of Cr on NR specific activity might be due to a reduced supply of NADH which might result from disorganization of chloroplasts, reduced rate of photosynthesis, respiration, NADH oxidation, or reduction in NO_3_^−^ supply to the site of the enzyme as a consequence of water stress induced by the metal and direct effect of heavy metals on protein synthesis [36]. Similar results have been observed by Rai et al. [37], where Cr toxicity resulted in the reduction of nitrate reductase activity through impaired substrate utilization in* Ocimum tenuiflorum*.

### 3.3. Nitrite Reductase

**Figure 3:**
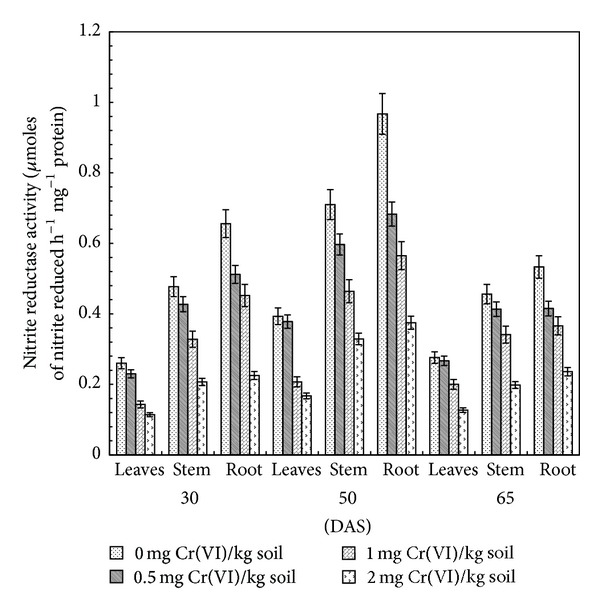
Effect of Cr(VI) on nitrite reductase activity in clusterbean plant parts (leaves, stem, and root) at different stages of growth.

NiR catalyzes the reduction of nitrite to ammonium in the second step of the nitrate assimilation pathway. A decrease in NiR specific activity (*μ*M of nitrite reduced h^−1^ mg^−1^ protein) was observed in leaves, stem, and roots of clusterbean plant treated with Cr(VI) in comparison to the control (Figure 3). Similar to nitrate reductase, specific activity of nitrite reductase also decreased with increasing level of Cr(VI) at different stages of growth. The maximum decrease in NiR specific activity as compared to control was 56.0, 57.6, and 54.0% in leaves, 56.7, 53.6, and 56.6% in stem, and 64.1, 61.3, and 55.7% in root with 2.0 mg Cr(VI) kg^−1^ soil treated plants at 30, 50, and 65 DAS, respectively. In every treatment, NiR activity increased to maximum at 50 DAS and decreased thereafter at 65 DAS (Figure 3).

These findings are in agreement with previous reports [18]. The observed decrease in NiR activity may be due to either of reduced carbon fixation, NO_3_^−^ uptake by roots and low NO_3_^−^ translocation in the xylem which may lead to subsequent limitation in reducing power and/or low NO_3_^−^ availability to plants [38]. Gajewska and Skłodowska [39] have shown that a decline in NiR activity was associated with the reduced availability of NO_2_^−^ ions, which are considered to have originated primarily from the NR-catalyzed NO_3_^−^ reduction. Moreover, it has been shown that the accumulation of NH_4_^+^ within a cell in higher concentrations alters intracellular pH and osmotic balance, inhibits ATP synthesis and secondary growth, and causes nutrient deficiency and chlorosis [40]. Therefore, under 2.0 mg Cr(VI) kg^−1^ soil treatment, the increase in NH_4_^+^ content may be linked with decreased growth of clusterbean plants. The observed decrease in NiR activity may either be due to reduced carbon fixation or low nitrate translocation in the xylem and, subsequently, low nitrate availability in shoot or reduced uptake of nitrate by roots or limitation in reducing power [18].

### 3.4. Glutamine Synthetase

**Figure 4:**
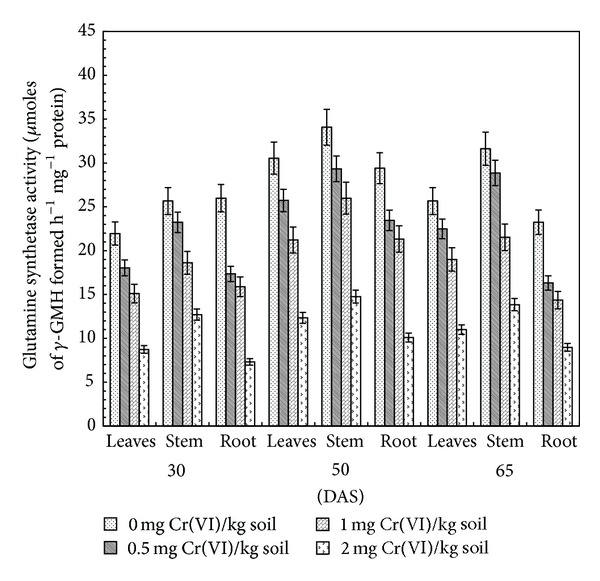
Effect of Cr(VI) on glutamine synthetase activity in clusterbean plant parts (leaves, stem, and root) at different stages of growth.

Ammonia is primarily assimilated through glutamine synthetase via GS/GOGAT pathway. Inorganic nitrogen could be assimilated by plants into the forms of glutamine and glutamic acid. GS is a key enzyme for nitrogen assimilation, which regulates nitrogen metabolism [41]. It is involved in the assimilation of ammonia derived either from nitrate reduction, N_2_ fixation, photo respiration, or asparagine breakdown. In the present study, the specific activity of GS was progressively decreased in leaves, stem, and root of clusterbean plants treated with increasing dosages of Cr(VI). The gradual decrease in GS specific activity (*μ*M of  *γ*-GMH formed h^*‒*1 ^mg^*‒*1^ protein) was observed at all stages of growth. Maximum decline in enzyme specific activity was recorded at 2.0 mg Cr(VI) kg^−1^ soil at all stages of growth. Compared to control, a 59.6, 56.7, and 65.7% decrease in its specific activity was observed at 50 DAS in leaves, stem, and root of plants treated with 2.0 mg Cr(VI) kg^−1^ soil, respectively. The enzyme specific activity increased from vegetative to flowering stage and then declined in different plant parts at the grain filling stage (i.e., 65 DAS) (Figure 4).

Inhibition of GS activity has also been described in barley seedlings [42]. The decreased availability of ATP and Mg^2+^ ions, which act as cofactors in various metabolic processes, may be the possible reason for the observed decrease in GS activity. Moreover, decreased activity in GS may also be responsible for increased NH_4_^+^ content as it has been shown that accumulation of high concentrations of NH_4_^+^ within a cell leads to nutrient deficiency and chlorosis, alters intracellular pH and osmotic balance, inhibits ATP synthesis, and eventually inhibits secondary growth [40]. Thus, it might be possible that supplementation of Cr led to an increase in NH_4_^+^ content and a decrease in GS activity. Several studies indicated degradation of nitrogenous compounds under heavy metal stress [36, 39].

### 3.5. Glutamate Synthase

**Figure 5:**
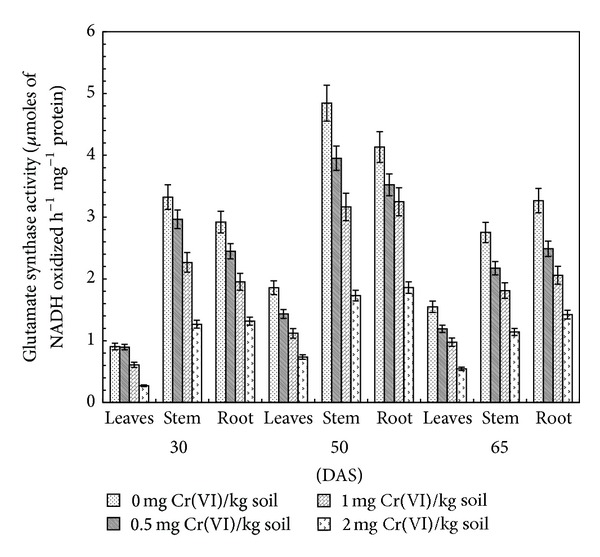
Effect of Cr(VI) on GOGAT activity in clusterbean plant parts (leaves, stem, and root) at different stages of growth.

The key enzyme involved in the* de novo* synthesis of glutamate is glutamate synthase, also known as glutamine: 2-oxoglutarate aminotransferase. The acidic amino acid is formed by the action of glutamate synthase, utilizing glutamine and 2-oxoglutarate. The reaction is a reductant-driven transfer of the amide amino group of glutamine to 2-oxoglutarate to yield two molecules of glutamate [43]. The specific activity of GOGAT (*μ*M NADH oxidized h^−1^ mg^−1^ protein) in various plant parts decreased significantly with increasing concentration of Cr(VI) at all stages of growth (Figure 5). Amongst the Cr(VI) levels, 2.0 mg kg^−1^ soil had a maximum adverse effect on its specific activity. At this concentration, the enzyme specific activity decreased by 70.0, 60.36, and 64.9% in leaves, 61.9, 64.3, and 58.5% in stem, and 54.9, 55.0, and 56.5% in roots as compared to control at 30, 50, and 65 DAS, respectively. The specific activity increased continuously in leaves, stem, and root up to 50 DAS and decreased thereafter at 65 DAS in each individual treatment (Figure 5). It is suggested that Cr may also induce premature senescence of plants through an increased level of NH_4_^+^ either through rapid reduction of NO_3_^−^ or enhanced proteolysis. Moreover, it has been shown that accumulation of NH_4_^+^ within a cell in higher concentrations leads to nutrient deficiency and chlorosis, alters intracellular pH and osmotic balance, inhibits ATP synthesis, and eventually inhibits secondary growth [40].

### 3.6. Glutamate Dehydrogenase

**Figure 6:**
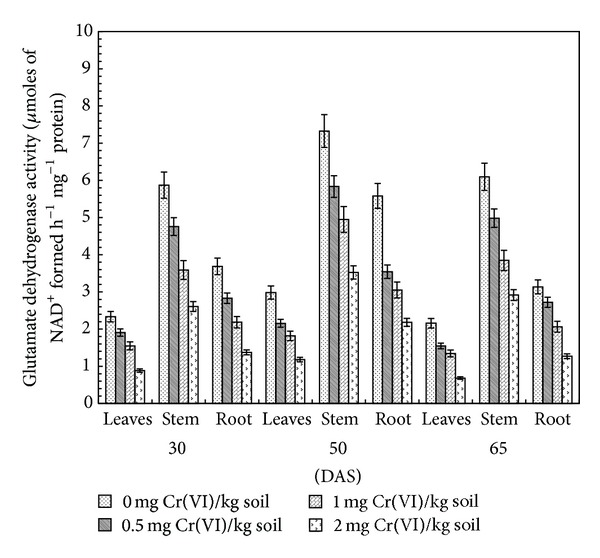
Effect of Cr(VI) on glutamate dehydrogenaseactivity in clusterbean plant parts (leaves, stem, and root) at different stages of growth.

In addition to GS and GOGAT, another enzyme potentially involved in ammonia metabolism is GDH. It can catalyze the reductive amination of 2-oxoglutarate and the reverse catabolic reaction of oxidative deamination of glutamate. It is located in the mitochondrial matrix where it is mainly responsible for glutamate catabolism under carbon and N-limiting conditions [44]. Glutamate dehydrogenase is present in the leaves, roots, and nodules in abundance [19]. Robinson et al. [45] have suggested that GDH activity is altered in response to shifts in carbon rather than nitrogen metabolism. Increasing the concentration of Cr(VI) from 0.0 to 2.0 mg Cr(VI) kg^−1^ soil resulted in a decrease in GDH specific activity (*μ*M NAD^+^ formed h^−1^ mg^−1^ protein) at different stages of growth in different plant parts. The specific activity decreased by 65.1, 60.5, and 68.4% in leaves, 55.6, 51.9, and 52.2% in stem, and 62.7, 60.9, and 59.5% in root as compared to the control at 30, 50, and 65 DAS, respectively, in 2.0 mg Cr(VI) kg^−1^ soil treated plants. In individual treatment, maximum enzyme specific activity was observed at 50 DAS followed by a decrease at 65 DAS. The stem exhibited higher GDH specific activity than leaves and root (Figure 6).

In the present study, results are in agreement with previous observations regarding Cr(VI) induced GDH inhibition in leaves, stem, and root of sorghum [18]. Burzyński and Buczek [46] studied the influence of Cu^2+^, Cd^2+^, Pb^2+^, and Fe^2+^ at various pH conditions (5.0, 6.0, and 7.0) on the uptake and assimilation of ammonia by cucumber seedlings and found that GS and NADH-GDH were also inhibited after 1 h of plant exposure to these metals. Joshi et al. [47] reported that the activity of GDH was increased in different plant parts of cowpea with Cr(VI) concentration up to 1 ppm and then decreased with further increase in it till 6 ppm. During the present study, GDH activity also decreased under Cr(VI) treatments. Heavy metals like Ni and Al treatments also impaired the nitrate assimilation process in rice seedlings by inhibiting the activities of key nitrogen assimilatory enzymes, that is, NR, GS, and GDH [48]. Evidence showed that GDH is not involved in ammonia assimilation, which occurs solely via the GS/GOGAT cycle. It was hypothesized that the primary role of GDH is the catabolism of glutamate to provide carbon skeletons for the TCA cycle function under conditions of carbon limitation, and consequently, this enzyme fulfills an important anapleurotic function linking carbon and nitrogen metabolism in higher plants [49].

## 4. Conclusion

In conclusion, our results indicate that Cr(VI) application adversely affected nitrogen metabolism by inhibiting the activity of these enzymes and growth of clusterbean plants, possibly as a result of its interference with photosynthetic pigments and key enzymes of nitrogen metabolism. The increased accumulation of Cr in plant parts negatively affected nitrogen metabolism. It may have further implications in designing and conducting similar studies as well as understanding the detailed mechanism of this toxicity and its removal thereafter.
